# Signal Conditioning for the Kalman Filter: Application to Satellite Attitude Estimation with Magnetometer and Sun Sensors

**DOI:** 10.3390/s16111817

**Published:** 2016-10-31

**Authors:** Segundo Esteban, Jose M. Girón-Sierra, Óscar R. Polo, Manuel Angulo

**Affiliations:** 1 Department of Computer Architecture and Automatic Control, Faculty of Physic Sciences, Complutense University of Madrid, Madrid 28040, Spain; gironsi@dacya.ucm.es; 2 Department of Computer Engineering, University of Alcala, Alcalá de Henares 28871, Spain; opolo@aut.uah.es; 3 Department of Space Programs, INTA, Torrejón de Ardoz 28850, Spain; angulo.manuel2@gmail.com

**Keywords:** attitude determination and control, magnetometer sensor, Sun sensors, Kalman filter, low cost satellites, quaternion, condition number

## Abstract

Most satellites use an on-board attitude estimation system, based on available sensors. In the case of low-cost satellites, which are of increasing interest, it is usual to use magnetometers and Sun sensors. A Kalman filter is commonly recommended for the estimation, to simultaneously exploit the information from sensors and from a mathematical model of the satellite motion. It would be also convenient to adhere to a quaternion representation. This article focuses on some problems linked to this context. The state of the system should be represented in observable form. Singularities due to alignment of measured vectors cause estimation problems. Accommodation of the Kalman filter originates convergence difficulties. The article includes a new proposal that solves these problems, not needing changes in the Kalman filter algorithm. In addition, the article includes assessment of different errors, initialization values for the Kalman filter; and considers the influence of the magnetic dipole moment perturbation, showing how to handle it as part of the Kalman filter framework.

## 1. Introduction

Most satellites have an attitude determination and control system (ADCS) [1]. In a space telescope the target of ADCS would be to obtain an accurate and stable position pointing to a desired target in the sky. The practical use of such a telescope may lead from time to time to changes in the target of interest, so the ADCS would execute adequate maneuvers to rotate the satellite. Efficiency criteria may demand fast maneuvers. Other types of mission, like data acquisition, Earth observation, communications, etc. would require other ADCS responsibilities and performance, probably more relaxed compared to the case of telescopes. Usually the ADCS employs sensors and actuators. High cost missions would have no issues with using very expensive components for the ADCS, like star trackers, laser gyroscopes, or high-precision wheels. However, the tendency now is to implement low-cost solutions. It is desirable to have the same performance, or better, than in the past, but using relatively cheap sensors and actuators. Therefore, efforts converge to devise algorithms, software, able to squeeze better performance out of what the on-board equipment can offer.

In more specific terms, if the ADCS was fitted with a star tracker and a gyroscope, it would directly provide the needed satellite orientation and angular velocity data, but in the case of low-cost satellites (LCS), cheaper, simpler, robust, and light weight sensors and actuators, would be preferred. Typically, the sensors used by LCS would be magnetometers and Sun sensors. The data provided by these sensors are vectors, which require processing for attitude estimation. This processing may magnify errors, and so a conditioning analysis would be recommended.

The usual framework for the study and design of satellite control is the state-space representation [2]. Actually, this representation was created in relation to artificial satellites, when they started to exist. A main event in the historical development of state-space theory has been the optimal state estimator system proposed by Kalman, which is known as the Kalman filter [3,4]. This estimator fits well with the needs of ADCS, when the state should be estimated from the measurements of some sensors not directly giving the state.

This article considers the use of the Kalman filter for ADCS based on magnetometers and Sun sensors. The attitude will be represented by quaternions [5], which offer important advantages related to avoiding singularities when the satellite rotates. 

During orbital motion, some ill conditioned problems can arise, causing the Kalman filter to malfunction. This is the main point addressed by this article, in which a solution is proposed.

Actually, the current authors were involved in the development, launching, and on-orbit monitoring of the INTA NS-1B nano-satellite. During the orbits, ill conditioned problems occurred, and we had to try several solutions. The most trivial was to add a kind of supervisor that switches from Kalman estimation to just propagated data, during the times when ill conditioning was expected. After that, a better alternative was developed, which is the main subject of the article.

The article has two main parts. One presents the proposed solution in general terms, useful for different satellite missions. The other focuses on an experimental context using the NS-1B satellite. This satellite is rotated by using magneto-torquers, which exert small torques and so the satellite rotating motions are smooth. 

**Figure 1:**
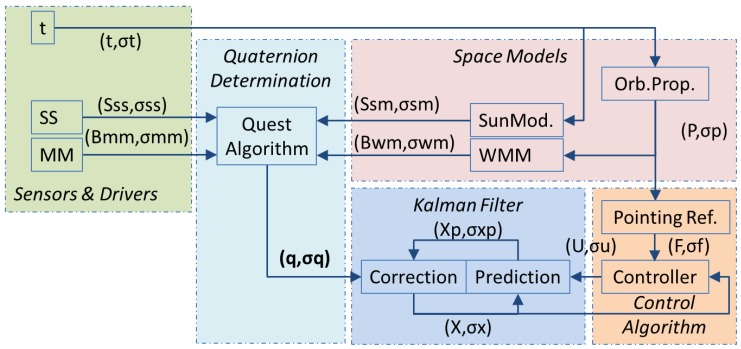
Attitude determination block diagram.

In order to present a functional perspective of the ADCS system considered in this article, Figure 1 shows a block diagram of the main parts of the system.

Each parenthesis in the diagram corresponds to signals and their noises. The diagram includes five sections:

- Sensors & Drivers: It gives the direction vector of the Earth’s magnetic field (Bmm, σmm) and the direction vector of the Sun (Sss, σss). The vectors are measured in Satellite Body Reference Frame (SBF). In addition, the ADCS should know the system time (t, σt).
- Space Models: Based on a set of mathematical models, namely a model of orbital propagation [6], a model of Earth magnetic field [7] and an orbital model of Earth [8], the ADCS calculates the orbital position (P, σp), a direction vector of the Sun (Ssm, σsm) and a direction vector of the Earth’s magnetic field (Bwm, σwm). The vectors Ssm and Bwm should be referenced to the Earth Centered Inertial Frame (ECI) [8].
- Quaternion Determination: The quaternion (q, σq) that describes the satellite orientation would be computed from measured vectors and calculated vectors. It will be shown in this article that ill conditioned problems can appear.
- Kalman Filter: In order to estimate the system state, the Kalman filter proceeds iteratively, with two steps in each iteration. The first step is prediction of state (Xp, σxp), and the second step is correction of that prediction (X, σx). The correction is based on the quaternion just determined at the time of correction.
- Control Algorithm: The attitude controller should generate a control signal (U, σu) for the actuation. The controller takes into account the estimated state (X, σx) and a reference signal (F, σf).

There is abundant literature covering the fundaments of satellite attitude control [9], the attitude representation [10], or the use of Kalman filter for attitude estimation [11,12]. In one of the first papers [13] not using gyros for attitude estimation, the authors commented that gyros are generally expensive and are often prone to degradation or failure. This article proposed a new algorithm different from the Kalman filter.

In the absence of gyros, the determination of angular velocity becomes a problem. In certain conditions, it could be inferred from other types of sensors. For instance, in [14] an inertial measurement unit (IMU) based on accelerometers was chosen; the article discusses observability issues and presents an extended Kalman filter (EKF) solution. Similarly, [15] introduced nonlinear observers using EKF and sensor information on attitude Euler angles.

New technologies, like micro electro-mechanical systems (MEMS) are attracting attention for its use on low-cost satellites. A distributed redundant accelerometer system, combined with an unscented Kalman filter (UKF) for angular rate estimation is proposed in [16].

The scenario considered in this article is also gyro-less. The estimation of angular velocity is possible by using an observable attitude representation with quaternions, described in the third section. In the fourth section, the focus is put on the ill conditioning problem that appears at the hour of quaternion determination. The problem is analyzed and then a solution is proposed. An important point is that the question is centered at the interface between sensors and the Kalman filter, and not the Kalman filter itself. Therefore, no modification of the Kalman filter is needed, and so any type of Kalman filter, EKF, UKF, etc., can still be used. The fifth section considers in particular the application to the NS-1B satellite, with experimental details. In addition to the contributions on observability and overcoming ill conditioned situations, the article includes an assessment of the importance of several types of errors present in the ADCS.

## 2. Background

It is convenient for the objectives of this article to summarize beforehand some background theory, concerning in particular quaternions and the Kalman filter. Both topics will be combined in the next sections for satellite attitude estimation.

### 2.1. Attitude Representation with Quaternions

The satellite attitude can be represented with a direction cosine matrix (DCM), which has the following expression using Euler angles:

(1) $$\mathrm{DCM}=\left[\begin{array}{ccc} \cos\left(\beta \right)\cos\left(\alpha \right) & -\sin\left(\beta \right) & -\cos\left(\beta \right)\sin\left(\alpha \right)\\ \sin\left(\beta \right)\cos\left(\alpha \right) & \cos\left(\beta \right) & -\sin\left(\beta \right)\sin\left(\alpha \right)\\ \sin\left(\alpha \right) & 0 & \cos\left(\alpha \right) \end{array}\right].$$

This representation is quite inefficient for attitude estimation, since it requires the estimation of nine parameters (the nine entries) whereas the attitude has only three degrees of freedom. 

It would be more advisable, for attitude estimation, to represent the attitude in terms of quaternions, $\bar{q}=\left(q_{0},q_{1},q_{2},q_{3}\right)$, because only four parameters are involved. The quaternion is defined as the sum of a scalar, $q_{0}$, and a vector, q, [5,17]:

(2) $$\bar{q}=q_{0}+q_{1}\cdot i+q_{2}\cdot j+q_{3}\cdot k=q_{0}+q$$

Quaternions represent a rotation by a rotational angle around an axis. This axis need not to be any of the axes *x*, *y* or *z*. In aerospace context, normalized quaternions with $q_{0}=\cos\left(\frac{\alpha }{2}\right)$ and $q=e\cdot \sin\left(\frac{\alpha }{2}\right)$ are used (*e* is the rotational axis). The multiplication of a vector, v, by a quaternion, $\bar{q}$ is a quaternion defined as:

(3) $$\bar{q}⊗v=-q\cdot v+q_{0}\cdot v+q\times v$$

Quaternions can be used as rotation operators. The quaternion operator will rotate a vector into another vector as follows:

(4) $$w=\bar{q}⊗v⊗{\bar{q}}^{*}$$

where ${\bar{q}}^{*}$ is the complex conjugate of $\bar{q}$. See [5,17] for more details on quaternion algebra.

The attitude of a satellite can be represented by the rotation of the satellite body frame relative to the inertial frame. Hence, it can be represented by a quaternion. When dealing with satellite rotations using matrices, difficulties related with singularities arise. A main advantage of quaternions is that this kind of problems is avoided.

### 2.2. State Variable Models

The dynamics and control of satellites are typically studied with state variables. The standard continuous-time state-variable model for linear multivariable systems is:

(5) $$\dot{x}\left(t\right)=A\cdot x\left(t\right)+B\cdot u\left(t\right)$$

(6) y(t)=C·x(t)+D·u(t),

where *x* is the state vector, *y* the output vector, u the input vector, and *A*, *B*, *C*, and *D* are matrices. Equation (5) describes the state dynamics, and Equation (6) the system output.

In order to use digital processing, equations should be discretized. The simplest way is the Euler’s approximation for the derivative:

(7) $$\frac{d*}{dt}\approx \frac{*\left(k\right)-*\left(k-1\right)}{T},$$

where *T* is the sampling period.

The discrete state-space representation of the linear system would have the form:

(8) x(k+1)=F·x(k)+G·u(k)

(9) y(k)=C·x(k)+D·u(k),

where F=(I+T·A) and G=T·B. In most systems, *D* is zero.

Based on Equations (8) and (9) the corresponding observability matrix would be:

(10) $$O=\left[\begin{array}{c} C\\ C\cdot F\\ \vdots \\ C\cdot F^{n} \end{array}\right],$$

The linear system is observable if the rank of *O* is equal to the dimension of the state vector.

### 2.3. Kalman Filter

The Kalman filter can be regarded as an optimal version of the Luenberger observer for noisy conditions. The mission of an observer is to estimate the states of a system based on inputs and outputs along time. The Kalman filter can be stated in the framework of discretized multivariable linear systems, departing from the following state space model:

(11) x(k)=F·x(k−1)+G·u(k−1)+w(k−1)

(12) y(k)=C·x(k)+v(k),

where w(k) is process noise, with variance *Q*(*k*); v(k) is measurement noise, with variance *R*(*k*). Both noises are zero-mean white noises. *F*, *G* and *C* are matrices. 

The prediction step of the Kalman filter would be:

(13) $$\hat{x}(k|k-1)=F\cdot \hat{x}\left(k-1\right)+G\cdot u\left(k-1\right)$$

(14) $$\hat{y}(k|k-1)=C\cdot \hat{x}(k|k-1),$$

where the hat was used to represent estimation.

The Kalman procedure uses a state covariance matrix, *P*, which is updated as follows:

(15) $$P(k|k-1)=F\cdot P\left(k-1\right)\cdot F^{T}+Q\left(k-1\right).$$

After the prediction step it comes a second, correction step that considers the last output measurement y(k):

(16) $$\hat{x}\left(k\right)=\hat{x}(k|k-1)+K\left(k\right)\cdot \left[y\left(k\right)-\hat{y}\left(k|k-1\right)\right],$$

where $\hat{x}\left(k\right)$ is the estimated state, and *K*(*k*) is computed as follows:

(17) $$K\left(k\right)=P\left(k|k-1\right)\cdot C^{T}\cdot {\left[C\cdot P\left(k|k-1\right)\cdot C^{T}+R\left(k\right)\right]}^{-1}.$$

The correction step also includes a modification of the matrix *P*:

(18) $$P\left(k\right)=\left(I-K\left(k\right)\cdot C\right)\cdot P\left(k|k-1\right)\cdot {\left(I-K\left(k\right)\cdot C\right)}^{T}+K\left(k\right)\cdot R\left(k\right)\cdot K{\left(k\right)}^{T}.$$

## 3. Attitude Estimation with the Kalman Filter

In the case considered in this article, the satellite attitude is a state vector to be estimated from the measurements obtained with a Sun sensor and a magnetometer. Another source of information is a state vector model of the attitude dynamics. The Kalman filter is precisely conceived for state estimation on the basis of measurements and a dynamics model.

### 3.1. Attitude Behaviour

The attitude dynamics of a rigid satellite can be described with the following equation:

(19) $$\frac{d\omega }{dt}=J^{-1}\cdot \left(\tau -\omega \cdot J\cdot \omega \right)=-J^{-1}\cdot \Xi \left(\omega \right)\cdot J\cdot \omega +J^{-1}\cdot \tau ,$$

where:

- *ω* is the angular velocity vector of the satellite in body axes;
- *J* is the tensor of inertia;
- *τ* is in body axes the vector of torques applied to the satellite: it includes perturbations and control actions;
- $\Xi \left(\omega \right)=\left[\begin{array}{ccc} 0 & -\omega_{3} & \omega_{2}\\ \omega_{3} & 0 & -\omega_{1}\\ -\omega_{2} & \omega_{1} & 0 \end{array}\right]$.

As said before, for attitude estimation it is better to use quaternions. In this case, the attitude kinematics would be described as follows:

(20) $$\left[\begin{array}{c} {\dot{q}}_{0}\\ {\dot{q}}_{1}\\ {\dot{q}}_{2}\\ {\dot{q}}_{3} \end{array}\right]=\frac{1}{2}\cdot \Omega \left(\omega \right)\cdot \left[\begin{array}{c} q_{0}\\ q_{1}\\ q_{2}\\ q_{3} \end{array}\right],$$

where $\Omega \left(\omega \right)=[\begin{array}{cccc} 0 & -\omega_{1} & -\omega_{2} & -\omega_{3}\\ \omega_{1} & 0 & \omega_{3} & -\omega_{2}\\ \omega_{2} & -\omega_{3} & 0 & \omega_{1}\\ \omega_{3} & \omega_{2} & -\omega_{1} & 0 \end{array}]$.

### 3.2. Observability Issues

**Figure 2:**
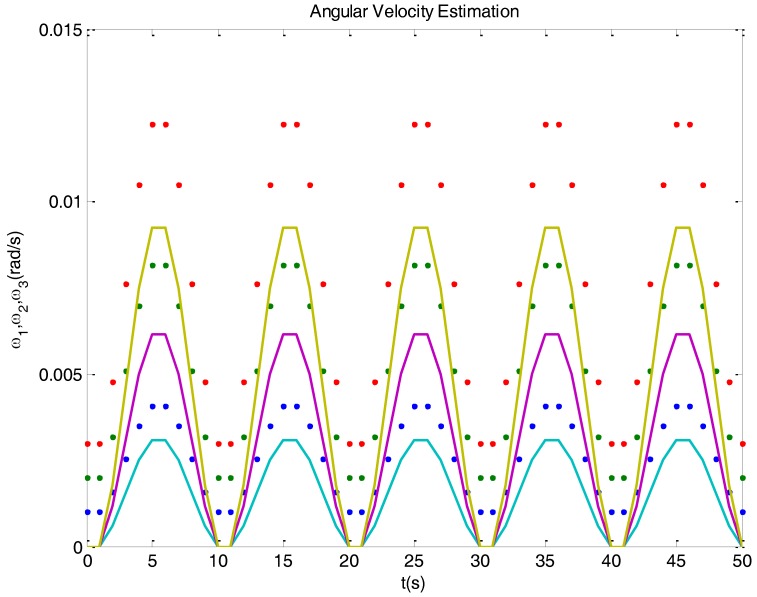
Real (dots) and estimated (solid) angular velocities in a simulated case, where a Kalman filter observer based on Equations (19) and (20) was used. The filter is not able to correctly estimate the velocity components, since the model used is not observable.

Let us advance that, if one uses a Kalman filter based on Equations (19) and (20) for angular velocity estimation, the result could be as depicted in Figure 2, which corresponds to the simulation of a typical satellite behavior. The estimation of real values, the dots, is represented with solid curves. The estimation notices value changes, but it maintains a bias. The problem is that the model used is not observable.

In our case, the system is nonlinear, but it can be considered as quasi-linear since the matrices in Equations (19) and (20) vary quite slowly. By using in Equation (20) the following equality is obtained:

(21) $$\left[\begin{array}{cccc} 0 & -\omega_{1} & -\omega_{2} & -\omega_{3}\\ \omega_{1} & 0 & \omega_{3} & -\omega_{2}\\ \omega_{2} & -\omega_{3} & 0 & \omega_{1}\\ \omega_{3} & \omega_{2} & -\omega_{1} & 0 \end{array}\right]\cdot \left[\begin{array}{c} q_{0}\\ q_{1}\\ q_{2}\\ q_{3} \end{array}\right]=\left[\begin{array}{cccc} q_{0} & -q_{1} & -q_{2} & -q_{3}\\ q_{1} & q_{0} & -q_{3} & q_{2}\\ q_{2} & q_{3} & q_{0} & -q_{1}\\ q_{3} & -q_{2} & q_{1} & q_{0} \end{array}\right]\cdot \left[\begin{array}{c} 0\\ \omega_{1}\\ \omega_{2}\\ \omega_{3} \end{array}\right],$$

**Figure 3:**
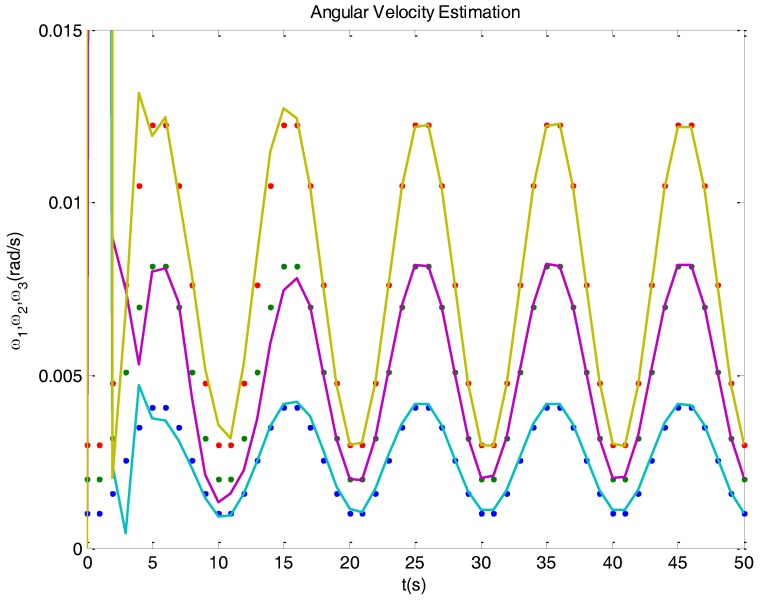
Real (dots) and estimated (solid) angular velocities in a simulated case, where a Kalman filter observer based on modified –observable– model was used. The filter now is able to correctly estimate the velocity components, since the model used is observable.

Some parts of the state dynamics model change (this is treated in more detail in Section 3.3), establishing connections between the angular velocity and the quaternion derivatives. In virtue of this change, the model becomes observable, so the angular velocity can be estimated from quaternions. As shown in Figure 3, the observer now quickly reacts against velocity biases, and obtains a correct estimation.

The Equation (21) can be found in some publications, like [13,17]. However, its use for observability reasons is not mentioned, probably because the context and focus of these texts.

The quaternions used for attitude representation are normalized to have modulus one. Hence, there is a constraint that can be expressed as follows:

(22) $$q_{0}=\sqrt{1-q_{1}^{2}-q_{2}^{2}-q_{3}^{2}},$$

It is known from practical applications that approximation and measurement errors could originate undesirable complex values of $q_{0}$; this should be prevented by error protection software. Based on Equation (22) a dimensional reduction of the state vector is possible, so the kinematics equation can be simplified as:

(23) $$\left[\begin{array}{c} {\dot{q}}_{1}\\ {\dot{q}}_{2}\\ {\dot{q}}_{3} \end{array}\right]=\frac{1}{2}\cdot \Psi \left(\bar{q}\right)\cdot \left[\begin{array}{c} \omega_{1}\\ \omega_{2}\\ \omega_{3} \end{array}\right],$$

where:

(24) $$\Psi \left(\bar{q}\right)=\left[\begin{array}{ccc} q_{0} & -q_{3} & q_{2}\\ q_{3} & q_{0} & -q_{1}\\ -q_{2} & q_{1} & q_{0} \end{array}\right].$$

Thanks to this simplification, the system state can be reduced to the following six components:

(25) $$x=\left[\begin{array}{c} \omega_{1}\\ \omega_{2}\\ \omega_{3}\\ q_{1}\\ q_{2}\\ q_{3} \end{array}\right]$$

### 3.3. Discretization

Using Euler’s approximation, one obtains from Equations (19) and (23) the following discrete state dynamics equation:

(26) $$x\left(k\right)=\left[\begin{array}{ccc} {I\;}_{3\times 3}-T\cdot J^{-1}\cdot \Xi \left(\omega \left(k\right)\right)\cdot J & & {0\;}_{3\times 3}\\ \frac{T}{2}\cdot \Psi \left(\bar{q}\left(k\right)\right) & & {I\;}_{3\times 3} \end{array}\right]\cdot x\left(k-1\right)+\left[\begin{array}{c} T\cdot J^{-1}\\ {0\;}_{3\times 3} \end{array}\right]\cdot \tau \left(k-1\right),$$

where ${I\;}_{3\times 3}$ is a 3 × 3 identity matrix and ${0\;}_{3\times 3}$ is a 3 × 3 matrix with all zeros. This equation gives the matrices *F* and *G* to be used by the Kalman filter (see Equation (9)).

The decisive point is that instead of all zeros, the first block of the second row in matrix *F* would contain non-zero components. This is the effect of using Equation (21), so observability is gained.

Assuming that the available sensors will yield the information required by the attitude quaternion, but not the angular velocities, the system output will only give the three quaternion components of Equation (25):

(27) $$y\left(k\right)=\left[\begin{array}{cc} {0\;}_{3\times 3} & {I\;}_{3\times 3} \end{array}\right]\cdot x\left(k\right).$$

This expression gives matrix *C* in Equation (12).

## 4. Quaternion Determination. Conditioning

In order to apply the Kalman filter correction step (Equation (16)), one needs to determine the attitude quaternion based on the available sensors. A usual case is to have a Sun sensor and a magnetometer, which yield a unit vector pointing to the Sun and a unit vector pointing to the magnetic North pole. This is a particular case of the so-called Wahba’s problem [18].

Suppose the measurement obtains two unit vectors $b^{1}$ and $b^{2}$ in the body frame. The same measurement is represented by two unit vectors $r^{1}$ and $r^{2}$ in an inertial frame. This type of measurements is usual when using Sun sensors, magnetometers, etc. The Wahba’s problem is to determine the quaternion operator that rotates vectors $r^{1}$ and $r^{2}$ to $b^{1}$ and $b^{2}$.

### 4.1. Quest

A simple method, called Quest [19], can be used for solving the problem. According with this method, the rotational quaternion ${\bar{q}}_{det}$ is obtained, from the already known vectors $r^{1}$, $r^{2}$, $b^{1}$ and $b^{2}$, as follows:

(28) $${\bar{q}}_{det}=\frac{\left[b^{2}\cdot r^{1}-b^{1}\cdot r^{2},\left(b^{1}-r^{1}\right)\times \left(b^{2}-r^{2}\right)\right]}{\sqrt{{\left(b^{2}\cdot r^{1}-b^{1}\cdot r^{2}\right)}^{2}+\left\|(b^{1}-r^{1}\right)\times {\left(b^{2}-r^{2}\right)\|}^{2}}},$$

where the denominator is in charge of quaternion normalization. From now on, the article will refer to the result of Equation (28) as the determined quaternion, while the Kalman filter will provide an estimated quaternion.

### 4.2. Problems Related to Singularities

**Figure 4:**
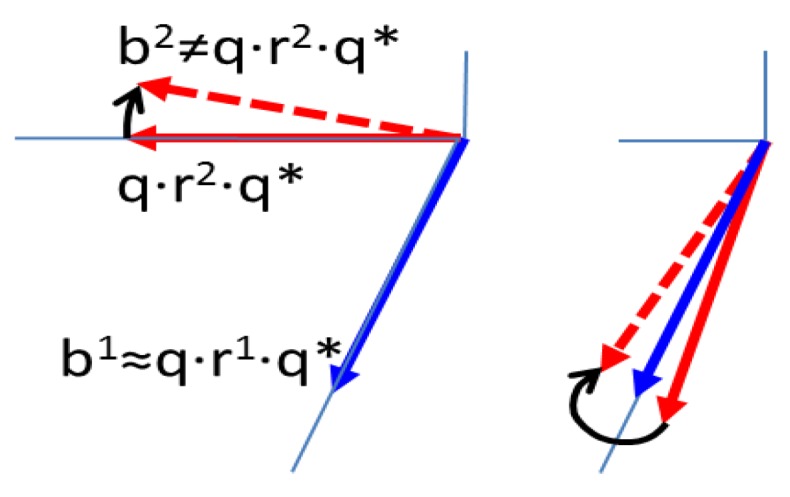
Good vector configuration (**Left**), and ill conditioned vector configuration (**Right**) for determining the attitude. The problem in the configuration on the right side is that a small error in a vector generates a considerable rotation error.

When the angle between the two vectors is small, tiny errors in the measurements cause large errors in the determination of the rotational quaternion. This singularity causes an ill conditioned situation, which would require a special treatment. Figure 4 illustrates this conditioning problem, showing on the left a good vector configuration, and on the right the ill conditioned vector configuration. 

**Figure 5:**
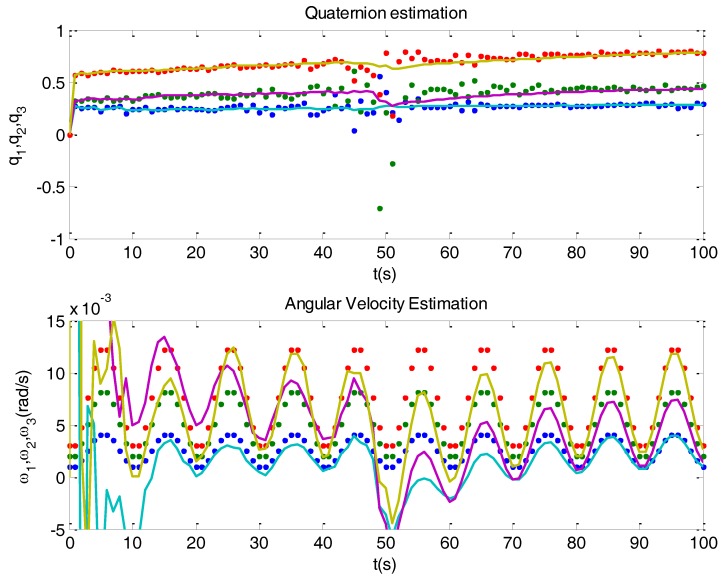
Top plot shows determined (dots) and estimated quaternion (solid) in a simulation with noisy observation vectors. Bottom plot shows simulated (dots) and estimated (solid) angular velocity. Notice the accommodation problem of the Kalman filter.

In order to visualize the problems related to singularities, a simulated example with just one singularity was devised. This is depicted in Figure 5 where, on top, there is a plot with the determined (dots) and the estimated (solid) quaternions, and at the bottom another plot with the simulated (dots) and estimated (solid) angular velocity. For illustrative purposes, a significant noise has been introduced in the observation vectors. The example departs at time 0 s with the two vectors being perpendicular, then gradually approaching so they get aligned at time 50 s, and then separate until becoming again perpendicular. There is a transient from time 0 s until the system stabilizes. What can be seen at time 50 s, when the singularity takes place, is a notable dispersion of the determined quaternion. This dispersion translates in amplified manner to the estimated angular velocity. The effects on the attitude control could be disastrous.

Another problem that can be noticed is the accommodation of the Kalman filter. Let us make a small digression on this. An important field where accommodation problems occur is system identification. The mission of system identification is to estimate system parameters from inputs and outputs. A possible objective would be to use system identification for adaptive control. For instance, when you drill with a machine, it would be convenient to have less aggressive action for soft materials, like clay, or more aggressive for stone. In the case of an aircraft you could expect dynamic changes as the fuel is being consumed and the weight decreases, and system identification would be useful for control adaptation. Recursive versions of identification are used in such cases. However there is a problem, along iterations the identification becomes less and less sensitive to changes. One could say that the identification sleeps. In particular, if any brisk and relevant change happens, it may go unnoticed (this would be dangerous with an aircraft). One of the solutions that have been proposed is to insert forgetting factors in the recursive algorithms. The problem of accommodation can also occur with the Kalman filter, when noise variances remain constant. Looking at the bottom plot in Figure 5, during the initial transient the Kalman filter converges in about 12 s; but the convergence after the singularity takes more, about 25 s. This drawback, that could be described as loss of agility, is due to accommodation, which is related to the small values acquired by the matrix *P*(*k*), (Equation (18)), causing low correction gains *K*(*k*), (Equation (17)).

### 4.3. A Solution Based on Conditioning

In order to wake up the recursive estimation procedure when there are relevant singularities, it is proposed to give a role to the condition number κ(q). Figure 4 helps to notice that conditioning problems may differ for one or another of the rotational degrees of freedom. For this reason it would be important to evaluate the condition number for each quaternion component. Moreover, typically the precision of sensors vary with the input direction, so it is convenient to evaluate the condition number with respect to each component of the measurement vector. Considering *q* as a vector function, the condition matrix would be the following Jacobian:

(29) $$\kappa \left(q\right)=\nabla q=\left[\begin{array}{ccccccccc} \frac{\partial q_{1}}{\partial b_{1}^{1}} & \frac{\partial q_{1}}{\partial b_{2}^{1}} & \frac{\partial q_{1}}{\partial b_{3}^{1}}\frac{\partial q_{1}}{\partial b_{1}^{2}} & \frac{\partial q_{1}}{\partial b_{2}^{2}} & \frac{\partial q_{1}}{\partial b_{3}^{2}}\frac{\partial q_{1}}{\partial r_{1}^{1}} & \frac{\partial q_{1}}{\partial r_{2}^{1}} & \frac{\partial q_{1}}{\partial r_{3}^{1}}\frac{\partial q_{1}}{\partial r_{1}^{2}} & \frac{\partial q_{1}}{\partial r_{2}^{2}} & \frac{\partial q_{1}}{\partial r_{3}^{2}}\\ \frac{\partial q_{2}}{\partial b_{1}^{1}} & \frac{\partial q_{2}}{\partial b_{2}^{1}} & \frac{\partial q_{2}}{\partial b_{3}^{1}}\frac{\partial q_{2}}{\partial b_{1}^{2}} & \frac{\partial q_{2}}{\partial b_{2}^{2}} & \frac{\partial q_{2}}{\partial b_{3}^{2}}\frac{\partial q_{2}}{\partial r_{1}^{1}} & \frac{\partial q_{2}}{\partial r_{2}^{1}} & \frac{\partial q_{2}}{\partial r_{3}^{1}}\frac{\partial q_{2}}{\partial r_{1}^{2}} & \frac{\partial q_{2}}{\partial r_{2}^{2}} & \frac{\partial q_{2}}{\partial r_{3}^{2}}\\ \frac{\partial q_{3}}{\partial b_{1}^{1}} & \frac{\partial q_{3}}{\partial b_{2}^{1}} & \frac{\partial q_{3}}{\partial b_{3}^{1}}\frac{\partial q_{3}}{\partial b_{1}^{2}} & \frac{\partial q_{3}}{\partial b_{2}^{2}} & \frac{\partial q_{3}}{\partial b_{3}^{2}}\frac{\partial q_{3}}{\partial r_{1}^{1}} & \frac{\partial q_{3}}{\partial r_{2}^{1}} & \frac{\partial q_{3}}{\partial r_{3}^{1}}\frac{\partial q_{3}}{\partial r_{1}^{2}} & \frac{\partial q_{3}}{\partial r_{2}^{2}} & \frac{\partial q_{3}}{\partial r_{3}^{2}} \end{array}\right],$$

where the components of the measurement vectors are: $b^{1}=\left[\begin{array}{ccc} b_{1}^{1} & b_{2}^{1} & b_{3}^{1} \end{array}\right]$, $b^{2}=\left[\begin{array}{ccc} b_{1}^{2} & b_{2}^{2} & b_{3}^{2} \end{array}\right]$, $r^{1}=\left[\begin{array}{ccc} r_{1}^{1} & r_{2}^{1} & r_{3}^{1} \end{array}\right]$ and $r^{2}=\left[\begin{array}{ccc} r_{1}^{2} & r_{2}^{2} & r_{3}^{2} \end{array}\right]$.

Based on the Quest method, Equation (28), the calculation of the derivatives in Equation (29) give the following simple results:

(30) $$\frac{\partial q_{i}}{\partial b_{i}^{1}}=\frac{\partial q_{i}}{\partial b_{i}^{2}}=\frac{\partial q_{i}}{\partial r_{i}^{1}}=\frac{\partial q_{i}}{\partial r_{i}^{2}}=0$$

(31) $$\frac{\partial q_{i}}{\partial b_{j}^{n}}={\left(-1\right)}^{n+j+1}\cdot \frac{\left(b_{\neq i\neq j\;}^{\neq n}-r_{\neq i\neq j\;}^{\neq n}\right)}{\left|q\right|}$$

(32) $$\frac{\partial q_{i}}{\partial r_{j}^{n}}={\left(-1\right)}^{n+j}\cdot \frac{\left(b_{\neq i\neq j\;}^{\neq n}-r_{\neq i\neq j\;}^{\neq n}\right)}{\left|q\right|}.$$

Now, the idea is to use the condition number κ(q) at each time *k*, for transmitting the variances from sensors to the determined quaternion:

(33) $$R\left(q\left(k\right)\right)=\left[\begin{array}{c} Rq_{1}\\ Rq_{2}\\ Rq_{3} \end{array}\right]=\kappa \left(q\left(k\right)\right)\cdot \left[\begin{array}{c} Rb^{1}\\ Rb^{2}\\ Rr^{1}\\ Rr^{2} \end{array}\right]\cdot \kappa^{T}\left(q\left(k\right)\right),$$

where $Rb^{n}$ and $Rr^{n}$ are vectors whose components are the variance of each component of the measurement vectors.

**Figure 6:**
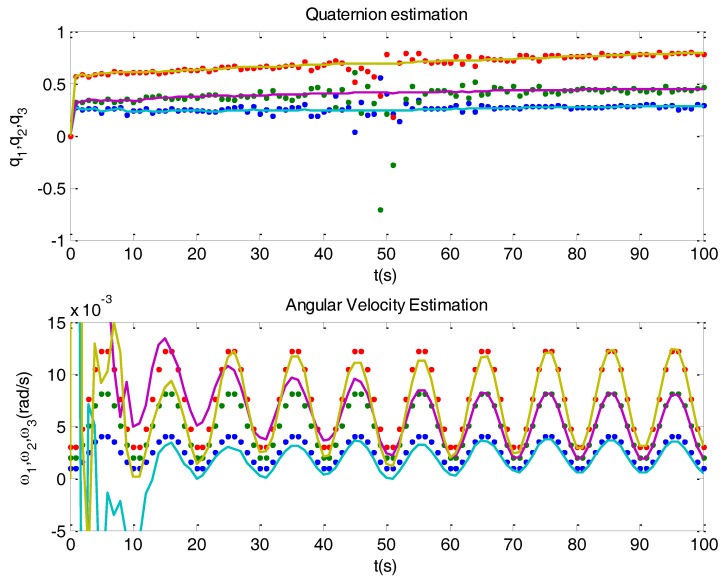
Result of the proposed calculation of quaternion variances, taking the same simulated example as in Figure 5. Top plot shows determined (dots) and estimated quaternion (solid). Bottom plot shows simulated (dots) and estimated (solid) angular velocity. In this case the filter isolates ill condition measurements and reduces accommodation.

**Figure 7:**
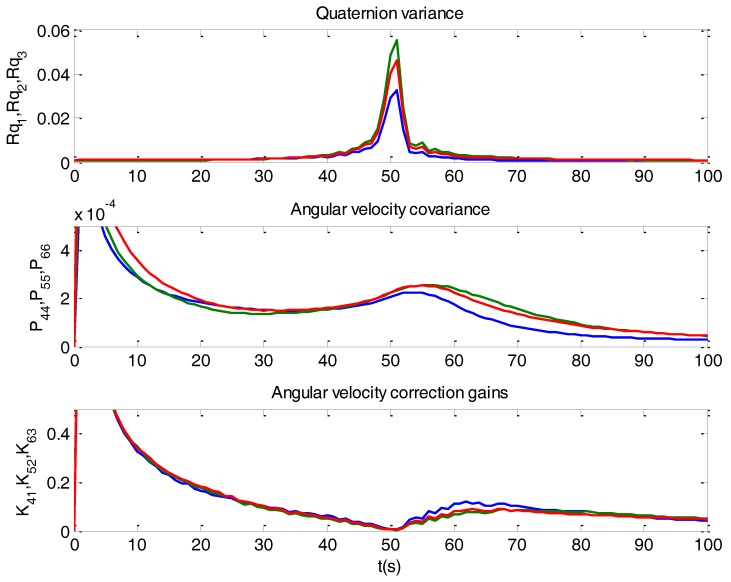
The plot on top shows the variances of the determined quaternion. The plot in the middle shows the main components of the covariance matrix of Kalman filter for the angular velocity. The plot at the bottom shows the main correction gains of Kalman filter for the angular velocity.

Figure 6 shows the results obtained when using the variances as computed in Equation (33). Compare with Figure 5. When a singularity occurs, the filter operates cautiously, with limited correction. It relies more on the model, acting mainly as a propagator. Since this fact causes an increasing of the covariance matrix *P*(*k*) in each iteration, correction gains increase after singularities, and so filter accommodation is avoided. Figure 7 depicts the mechanisms when a singularity takes place. 

It can be noticed in Figure 7 that when a singularity happens the variances of quaternion components jump two orders of magnitude (plot on top). Each of these components has a different variance, since alignment has a different influence on each of them. The high variance values make the Kalman correction gains become almost zero (plot at the bottom). Then the filter works in propagation mode, and the covariance matrix increases (middle plot). This increase causes the correction gains also to increase after the singularity.

## 5. Aspects of the Application to a Satellite

The topics treated in this article were motivated by our experience with the INTA NS-1B satellite, now in orbit. The satellite’s ADCS is based on Sun sensors, magnetometers and magneto-torquers. The experimental measurements that have been obtained have significant relevance for the practical setting of the Kalman filter. In particular, an important aspect, not usually mentioned in the literature, is a realistic specification of noise covariances. Another aspect, which can be observed from measurements, is the influence of the magnetic dipole moment (MDM). The MDM is the most important perturbation suffered by the ADCS. In this section, the MDM will be integrated in the system model, and so in a natural way it becomes part of the proposed estimation method. The section starts with a description of the NS-1B satellite. Then it pays attention to noises and their influence on attitude estimation precision. Finally, it focuses on MDM.

### 5.1. INTA Nanosat-1B Satellite

**Figure 8:**
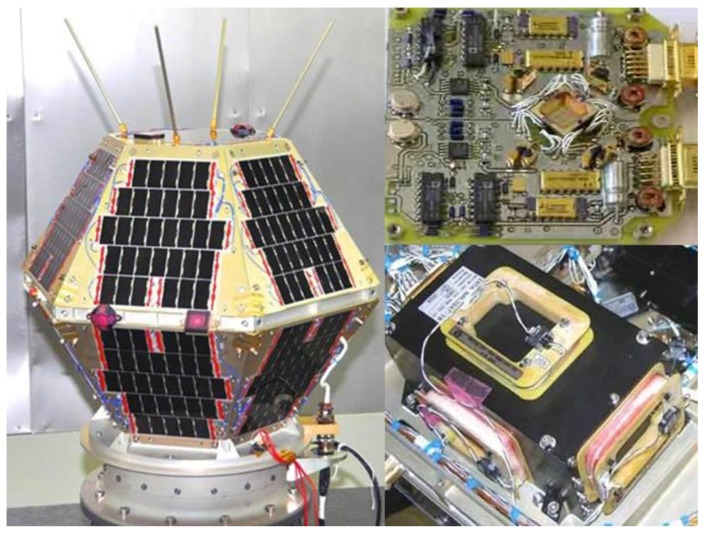
INTA NS-1B ADCS sensors and actuators. On the left hand side: Sun sensors (truncated pyramids) are on the equatorial border and on top panel of the satellite. On top right: The complete magnetometer; on bottom left: Three magneto-torquers attached to a box.

The INTA Nanosat-1B (NS-1B) [20], with a mass of 23.9 kg, was launched on July 2009; its orbit is LEO (615 km), heliosynchronous polar with very low eccentricity and LTAN 10:30. The satellite has an attitude determination and control subsystem (ADCS) based on the following sensors and actuators: one magnetometer (MM), three coarse Sun sensors (SS), and three magneto-torquers (MT). Both the sensors and actuators were made in-house. Figure 8 shows these sensors and actuators. 

The satellite is used for deferred communications. It is required that the absolute pointing error must be less than 10°. The attitude determination has an important computational cost. Since the NS-1B satellite is physically symmetric and compact, solar and gravitational perturbations could be considered negligible. The magnetic perturbation is, instead, significant, since it is difficult to get a magnetically clean satellite. Indeed, the satellite was built with components not prone to magnetization, but in most satellites there is always some remnant magnetic dipole.

### 5.2. Measurement Noise and Sensors

#### 5.2.1. Magnetometer

**Figure 9:**
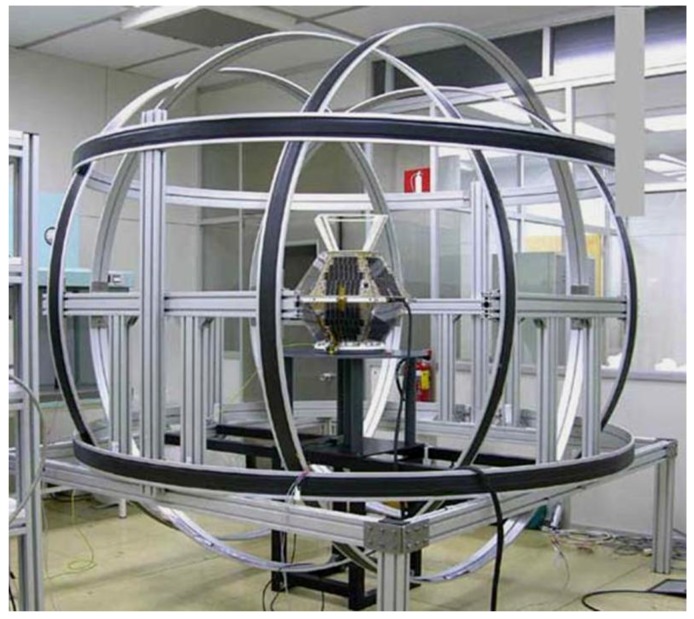
NS-1B Magnetometer calibration setup.

The magnetometer has been built in house by the INTA Opto-Electronics Lab [21]. It has been calibrated on ground using the experimental setting shown in Figure 9.

The core of the MM is a group of four magneto-resistors (MR) with suitable orientations. The fourth MR is included to obtain a fault tolerant sensor. The MRs must measure magnetic field intensity up to ±0.5 Gauss in the NS-1B orbit. The signals from the MR are transformed to the 0.10 V range, in order to be digitalized by 12 bit analog/digital converters (ADC). Because the field may be positive or negative, the set point is placed on the middle of the scale, 5 V. The satellite calibration offsets can reach ±2.5 V. In summary, one can use a range of 2.5 V for the measurement of up to 0.5 Gauss; in other terms, ¼ of the ADC scale: 10 bits. Therefore, the maximum digital precision for B direction measurement would be:

(34) $${\mathrm{MM}}_{\mathrm{DigitalPrecission}}=\frac{1/4}{2^{12}}=9.7656\times 10^{-4}\;\mathrm{units}.$$

It has been confirmed with telemetry once the NS-1B was in orbit, that the noise in the magnetometer signal was of that order.

Concerning the direction measurement accuracy, due to both the magnetic sensor and the magnetic field model the errors are in the order of one degree. These errors have components of very low frequency, which are mainly due to the influence of the MDM on the magnetometer calibration and to the errors of the magnetic field model. 

In more detail, the errors of the magnetic field model include errors of the World Magnetic Model (WMM), and errors of the orbital propagator (which is used to compute the orbital position of the satellite).

It is not possible to eliminate with the standard Kalman filter the very low frequency errors. Therefore, they should be taken into account when evaluating the accuracy. Coming back to Equation (34), in a normal probability distribution a ±3σ margin is equivalent to a confidence interval of 98%. Then, one obtains the following measurement noise variance:

(35) $$R_{MM}={\left(9.7656\times 10^{-4}/3\right)}^{2}=1.0596\times 10^{-7}.$$

#### 5.2.2. Sun Sensors

**Figure 10:**
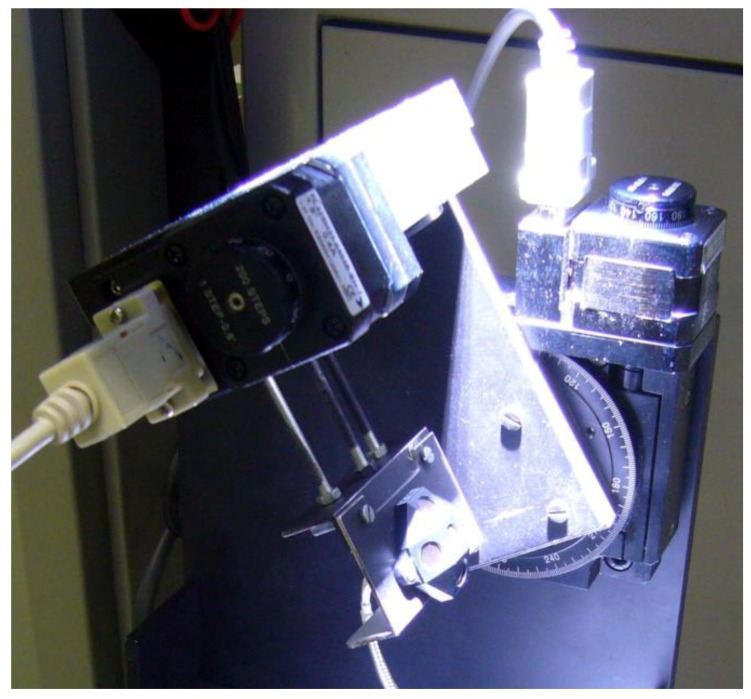
Experimental setup to evaluate Sun sensor drivers. In this case the Sun sensor is suffering shadow and albedo effect.

Each Sun sensor (SS) uses five gallium arsenide photocells with a coverglass for the space environment. At least three cells should be used to be able to determine the Sun direction. In our case the performance has been improved by using five cells, which provide redundant information (this is especially valuable in the case of satellites) [22]. This redundant information can be used by the sensor drive for failure detection and for perturbation isolation (examples of perturbations are the Earth’s albedo, or the shadows from antennae). Figure 10 shows experimental tests of sensor drivers.

The response of a solar cell, in terms of current, depends on the captured light irradiance. This irradiance is described by the Lambert’s cosine Law. The current generated by the cells are lineally translated to voltage by simple electronics. The measurement is only in one sense and then it is possible to use ¾ of the ADC range:

(36) $${\mathrm{SS}}_{\mathrm{DigPrec}}=\frac{3/4}{2^{12}}=3.2552\times 10^{-4}\;\mathrm{units}.$$

Although it is not possible to benefit from this higher precision, SS measurements are strongly perturbed by albedo, especially at the beginning of eclipse. The albedo could introduce errors in the order of two degrees, because of the driver software internal working. The perturbations due to shadows or albedo do not behave like white noise; instead they have low frequency components that cannot be eliminated by the Kalman filter. Like before, considering a ±3σ margin, one obtains the following measurement noise variance:

(37) $$R_{SS}={\left(3.2552\times 10^{-4}/3\right)}^{2}=1.1774\times 10^{-8}.$$

Once one has obtained $R_{MM}$ and $R_{SS}$ it is possible to use Equation (33) for computing the R matrix of the determined quaternion. This is the matrix to be used in Equations (17) and (18).

### 5.3. Process Noise

#### 5.3.1. Input and Perturbation Noise

Depending on the orbit and the satellite equipment, the process noise would have specific characteristics. For instance, and interplanetary satellite would not suffer from the Earth’s magnetic field, after taking enough distance in space. Our particular experience is directly related with the nano-satellite INTA NS-1B, in low Earth orbit (LEO). The orbit chosen goes from pole to pole, circling the Earth each 90 min. Although every effort is made to avoid it, the fact is that satellites have some remnant magnetic dipole, which causes the most important of the process perturbations as this dipole interacts with the Earth’s magnetic field. By data monitoring while the satellite is orbiting, it is possible to estimate the values of this perturbation. The NS-1B attitude actuation is made with magneto-torquers (MT). To give an idea of the importance of the satellite magnetic dipole, Table 1 shows magnitudes of actuation and perturbation torques.

During the design of the ADCS, the value of the magnetic dipole moment that can be generated by the actuators, MagDip_MT_, has been decided considering the satellite inertia. The MDM of the satellite, MagDip_SAT_, was first been estimated on ground for checking whether it can be cancelled by the actuators; however, it must be estimated again once in orbit. Since the characteristics of the orbit were known, the maximum value of the Earth’s magnetic field (as felt by the satellite), |B_max_|, is known. With this information it is possible to evaluate the maximum values of actuation and perturbation torques. Concerning the actuation signal, a 1% of its maximum value would be assigned to error, being Q_MT_ its variance. With respect to the MDM, it would be not modeled by now, so a 100% of its magnitude would be assigned to error, being Q_PER_ its variance.

#### 5.3.2. Discretization Noise

Let us take in consideration the real importance of having used the Euler’s approximation Equation (7) to obtain the model for the discrete Kalman filter. It is also convenient, at least for comparison purposes, to assess the importance of the process noise modeled in this same equation.

Consider the following Taylor expansion:

(38) $$\omega \left(k+1\right)=\omega \left(k\right)+T\cdot \frac{d\omega }{dt}+\frac{1}{2}\frac{d^{2}\omega }{dt^{2}}\cdot T^{2}+o\left(T^{3}\right)+\cdots$$

(39) $$q\left(k+1\right)=q\left(k\right)+T_{det}\cdot \frac{dq}{dt}+\frac{1}{2}\frac{d^{2}q}{dt^{2}}\cdot T_{det}^{2}+o\left(T_{det}^{3}\right),$$

The term in $o(T^{3}$) and the next terms would be negligible since the satellite would have smooth motions, which is not the same with airplanes or missiles. Thus, the term in $o(T^{2}$) could be taken as error bound. Recalling Equations (19) and (20), and considering the worst case, it is possible to obtain:

(40) $$\left|\frac{d^{2}\omega }{{dt}^{2}}\;\right|\leq \left({\left|\frac{d\tau }{dt}\right|}_{max}+{\left|\frac{d\omega }{dt}\times I\times \omega \right|}_{max}+{\left|\omega \times I\times \frac{d\omega }{dt}\right|}_{max}\right)/J_{min}=2.0311\times 10^{-8}$$

(41) $$\left|\frac{d^{2}q}{{dt}^{2}}\right|=\frac{1}{2}\cdot \left|\Omega \left(\frac{\tau_{MTmax}}{J_{min}}\right)\right|\cdot \left|\dot{q}\right|\leq \frac{1}{2}\times \frac{2.5\cdot 10^{-5}}{0.54}\times \frac{1}{2}\times 10^{-3}\times 1\leq \;1.1574\times 10^{-7}.$$

It can be seen, then, that the error committed by using Euler is very small. However, during iterations Equation (22) could yield undesirable complex results, which can be avoided by repeated quaternion normalization.

These errors originated by the Euler’s approximation can be regarded as white noise with 3σ amplitudes, and so with variances:

(42) $$Q\omega_{DIS}={\left(\frac{2^{2}}{2}\;2.0311\times 10^{-8}/3\right)}^{2}=1.8335\times 10^{-16}$$

(43) $$Qq_{DIS}={\left(\frac{2^{2}}{2}\;5.5728\times 10^{-6}/3\right)}^{2}=1.3803\times 10^{-11}$$

As a simplification, one could assume that all perturbations are white noise, with variances *Q_DIS_*, *Q_MT_* and *Q_PER_*. In total, the variance of the process noise, *Q*, would be:

(44) $$Q\left(k\right)=Q_{DIS}\left(k\right)+G\cdot \left(Q_{MT}\left(k\right)+Q_{PER}\left(k\right)\right)\cdot G^{T}.$$

**Figure 11:**
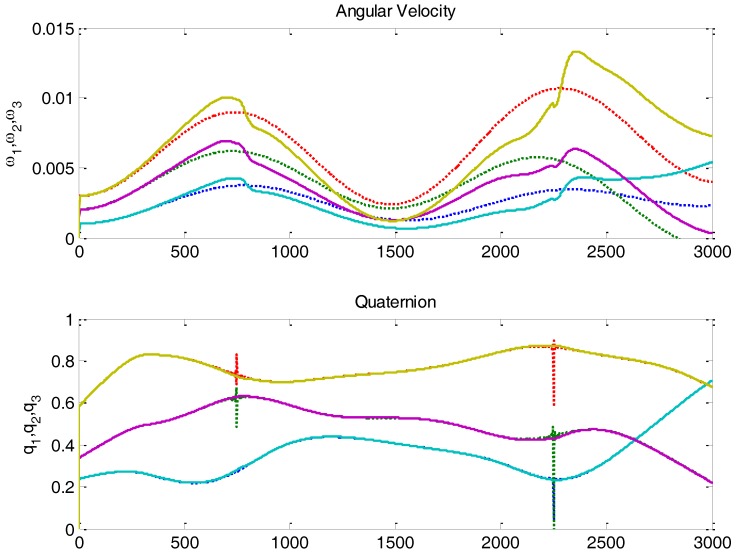
NS-1B attitude estimation using Kalman filter without magnetic perturbation modelling. The angular velocity estimation (solid line) does not converge to simulation due to magnetic dipole perturbation.

Now, using the values of variances just evaluated, it is possible to configure a Kalman filter for the attitude estimation of the NS-1B satellite. Figure 11 shows this attitude estimation during half an orbit, from pole to pole, in which there are two Sun and ***B*** alignment singularities.

The experiment shown in Figure 11 has done by simulation. As shown in the plot on top, the estimation of angular velocity initially converges, but then it is not able to track the true (simulated) values. Even before the singularity, the estimation starts to diverge. This is due to the fact that MDM is not white noise, having instead a significant low frequency component. The filter is not able to counteract it, and therefore estimation deviations occur. Luckily, this perturbation can be modeled and added to the Kalman filter equations.

### 5.4. Magentic Dipole Estimation

In order to include the MDM perturbation into the Kalman filter framework, one considers the torque caused by this perturbation:

(45) $$\tau_{PER}=MagDip_{SAT}\times B=-\Omega \left(B\right)\cdot MagDip_{SAT}$$

This equation connects *MagDip_SAT_* with the angular velocity. The magnetic dipole should be estimated in flight. The idea is to make it part of the complete state to be estimated. A new state dynamics equation is written as follows:

(46) $$x\left(k\right)=\left[\begin{array}{ccc} {I\;}_{3\times 3}-T\cdot \;J^{-1}\cdot \Xi \left(\omega \left(k\right)\right)\cdot J & {0\;}_{3\times 3} & -T\cdot J^{-1}\cdot \Omega \left(B\left(k\right)\right)\\ \frac{T}{2}\cdot \Psi \left(\bar{q}\left(k\right)\right) & {I\;}_{3\times 3} & {0\;}_{3\times 3}\\ {0\;}_{3\times 3} & {0\;}_{3\times 3} & {I\;}_{3\times 3} \end{array}\right]\cdot x\left(k-1\right)+\left[\begin{array}{c} T\cdot J^{-1}\\ {0\;}_{3\times 3}\\ {0\;}_{3\times 3} \end{array}\right]\cdot \tau_{MT}\left(k-1\right)$$

where the input is the torque generated by the actuators.

**Figure 12:**
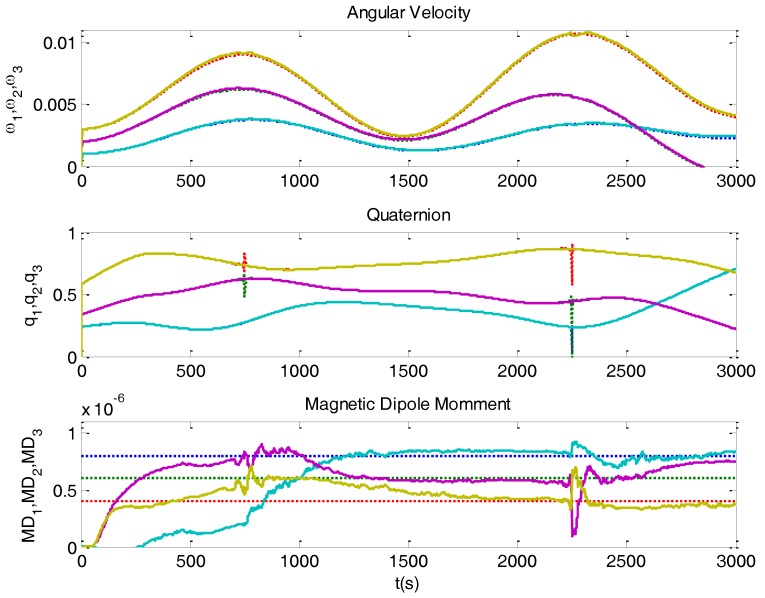
NS-1B attitude estimation (solid line) using Kalman filter including dipole moment estimation. It can be noticed that there are some *MDM* observability problems, causing divergences in some time intervals.

Thanks to the new degree of freedom, the state estimation is now able to obtain good results, as depicted in Figure 12.

The Ω(B(k)) matrix has zeros in its diagonal, hence preventing the system from being completely observable. The maximum observability degree that could be obtained is 8, therefore some components of *MagDip_SAT_* would be not observable. Fortunately, the magnetic field (as seen by the satellite) has two complete rotations per orbit; this makes possible to estimate different dipole components in different parts of the orbit, as shown in Figure 12 (curves that converge to true values). 

A practical aspect that deserves a comment is the Kalman filter initialization. It is necessary to assign priorities to initial convergences: quaternions should be the first to converge, then the angular velocity (which is estimated from quaternions), and finally the MDM since it has a slower dynamics. Problems with interruptions of filter software must be avoided too [23]. 

## 6. Conclusions

The topic of satellite attitude estimation using Kalman filters has been widely treated in the aerospace literature. In large budget missions, expensive sensors like star trackers or gyroscopes are used, being easily integrated in a Kalman filter framework. However, there is a growing interest in low-cost missions with other types of sensors having moderate cost, low weight and small size. The integration of these sensors in the Kalman filter procedure is not so obvious, as it has been shown in this article. Problems of signal conditioning and state observability do arise.

In this article a series of aspects, not usually mentioned in the literature, like system observability, ill conditioned situations, and Kalman filter accommodation has been considered, in the context of quaternions for attitude representation. The article includes a new proposal that solves the identified problems, and that can be used in any Kalman filter version.

The proposed ideas have been applied in a real satellite now in orbit, with satisfactory results. Taking advantage of experimental information, an assessment of precision and errors that influence the performance of the Kalman filter was done. A specific treatment of the magnetic dipole moment perturbation, which is most important in LEO satellites, has been accomplished with good results.

It would be interesting for further work to examine how the use of gyros, even low-cost gyros, compares with using the devices considered in this article (Sun sensors and magnetometers). Future research will consider in more detail the accuracy of sensors, focusing on systematic errors.

## Figures and Tables

**Table 1:** Magnitudes of actuation and perturbation in the satellite NS-1B.

Variable	Magnitude	Error % | Variance
|MagDip_MT_|	0.5 A·m^2^	
|MagDip_SAT_|	0.02 A·m^2^	
|B_max_|	0.5 × 10^−4^ Teslas	
τ_MT_ ≤ |B_max_|·|MagDip_MT_|	2.5 × 10^−5^ N·m	3σ = 1% | Q_MT_ = 6.9444 × 10^−15^
τ_PER_ ≤ |B_max_|·||MagDip_SAT_|	1 × 10^−6^ N·m	3σ = 100% | Q_PER_ = 1.1111 × 10^−13^
